# Supplementary material for: On the Close Relatedness of Two Rice-Parasitic Root-Knot Nematode Species and the Recent Expansion of Meloidogyne graminicola in Southeast Asia
Source: Genes (Basel). 2019 Feb 25;10(2):175. doi: 10.3390/genes10020175 (PMC6410229; doi:10.3390/genes10020175)
Supplement: Supplementary file 1 [file genes-10-00175-s001.pdf]

Supplementary Information

On the close relatedness of two rice-parasitic root-knot nematode species and the recent expansion of *Meloidogyne graminicola* in Southeast Asia

Guillaume Besnard, Ngan ThiPhan, Hai Ho Bich, Alexis Dereeper, Hieu Trang Nguyen, Patrick Quénéhervé, Jamel Aribi and Stéphane Bellafiore

Supplementary information includes the following items:

Supplementary methods1. Reference genomes used to screen contaminants

Supplementary methods 2. Estimation of genome size and depth of sequencing

Figure S1. *k*-mers distribution of *M. graminicola* and *M. oryzae* genomes at *k* = 15, 19, 23 and 27

Figure S2. Comparative alignment of repeated elements composing the 65R/94R/63R mitogenomic region of *M. oryzae*, *M. graminicola* and *M. incognita*, respectively

Figure S3. Linearized representation of mitogenomes of *M. oryzae* and *M. graminicola*

Figure S4. Reduced-median network of *Meloidogyne graminicola* mitochondrial haplotypes including the information of heteroplasmic sites

Figure S5. Distribution of sequence polymorphisms between ribotype I and II along the whole nrDNA cluster of *M. graminicola* and *M. oryzae*

Figure S6. Phylogenetic relationships within *Meloidogyne* species based on ITS

Table S1.Origin and genome sequencing data summary generated in this study for each nematode isolate

Table S2. Accession numbers of mitochondrial genes of *M. hapla* and *M. floridensis*

Table S3. Diversity of mitochondrial genomes between and within the *Meloidogyne* species

Table S4. Position of SNPs, indels and heteroplasmic sites within the *M. graminicola* mitogenome among 13 isolates

Table S5. Assessment of the assembly completeness of available *Meloidogyne graminicola* genome and transcriptome

Table S6. Sequence identity between each type of homolog for the three nuclear regions investigated

Table S7. Sequencing depth of each type of homolog for the nuclear genomic regions TAA6 and ACC6 among 12 *M. graminicola* and two *M. oryzae* isolates

References

Supplementary methods

Supplementary methods 1. Reference genomes used to screen contaminants

Reference genomes used to screen contaminants include (1) NCBI RefSeq bacterial genomes (70,293 entries as of 8 December 2016), (2) NCBI GenBank fungal genomes (2,314 entries as of 4 May 2017), rice genomes (IRGSP-1.0_genome and nippon_ir64 from Schatz laboratory), human genome (GRCh38/hg38) and the mitogenomes presented in this work.

Supplementary methods 2. Estimation of genome size and depth of sequencing

k-mers are nucleotide sequences that can be extracted directly from sequencing reads by a sliding window of length *k*. For novel genome, *k*-mer analysis can reveal some insights in terms of structure and complexity. Hereafter, read length is denoted as *L*, total number of reads as *N*, haploid genome size as *G*, sequencing depth as *S*, and *D* as average homozygous coverage. We excluded *k*-mers with coverage out of range 5 to 1,000, denoted as *B*. Based on the hypothesized ploidy, *D* is determined from the average unique heterozygous λ. In our case, if *M. graminicola* is deemed as diploid and *M. oryzae* as triploid, the average homozygous coverage is 2λ and 3λ, respectively [1]. Repetitive homozygous *k*-mers, used to calculate repeat proportion, have coverage of more than 6 * λ. Considering heterozygosity rate as the probability of a heterozygous nucleotide, we can indirectly estimate it via the number of unique heterozygous *k*-mers. *G* and *S* are calculated as follows:

$$G = \frac{N * (L - K + 1) - B}{D}$$

$$S = \boldsymbol{\lambda} * \frac{L}{L - K + 1}$$

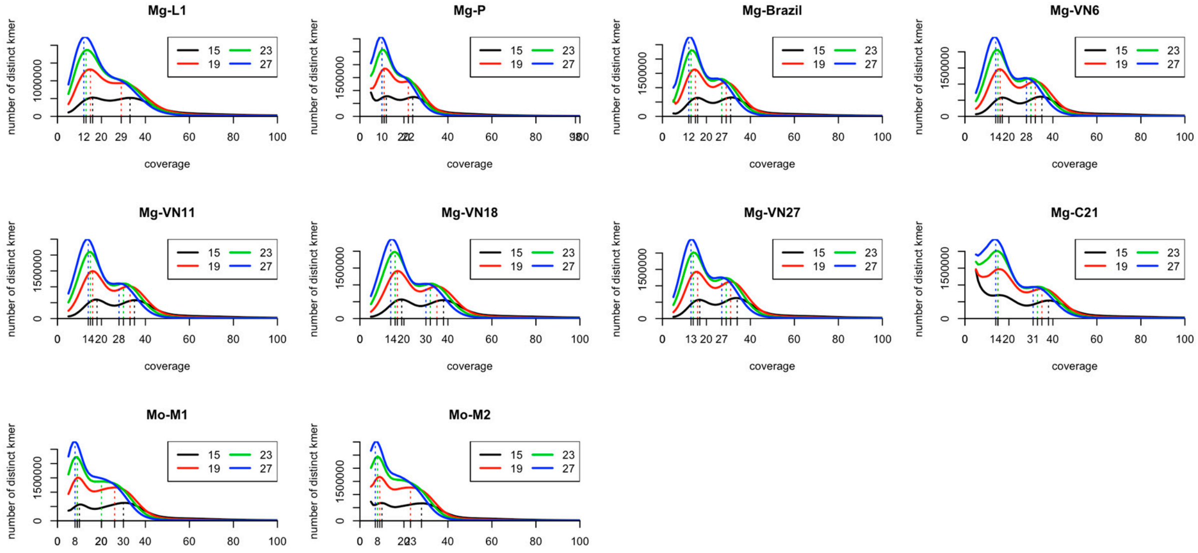

Figure S1. *k*-mers distribution of the *M. graminicola* and *M. oryzae* genomes at *k* = 15, 19, 23 and 27. Histogram curves for *k* = 27 (blue curve), *k* = 23 (green curve), *k* =19 (red curve) and *k* = 15 (black curve).

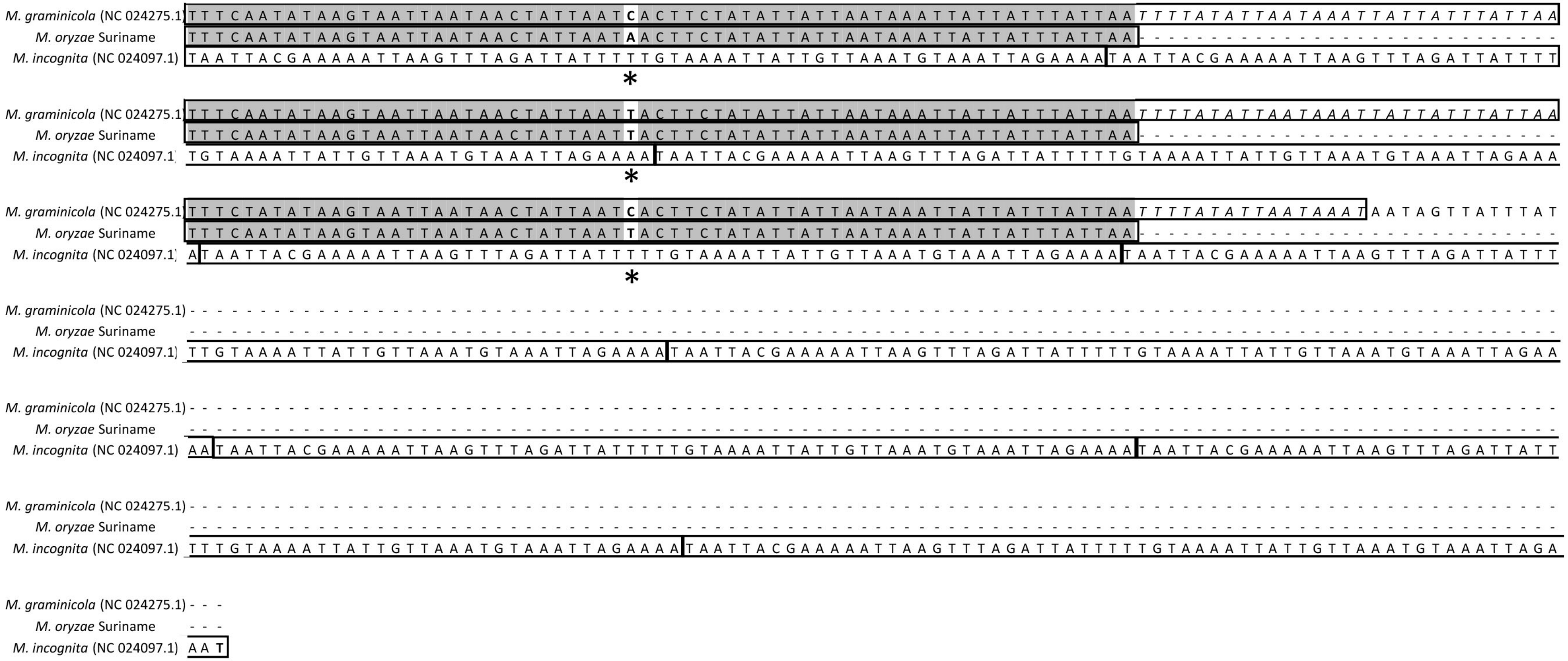

Figure S2. Comparative alignment of repeated elements composing the 65R/94R/63R mitogenomic region of *M. oryzae* (Mo, this study), *M. graminicola* (Mg, NC_024275.1) and *M. incognita* (NC 024097.1), respectively. The boxes indicate the repeated elements in each species. Three repeated 65-bp elements in *M. oryzae* overlap with 94R region in *M. graminicola* which includes two repeated 94-bp elements and one 81-bp element. At position 31 of the 94-bp element, a variable site (C, A or T) was found between repeats for all Mg and Mo populations. For the three repeats, Mg got respectively the nucleotides C-T-C and Mo A-T-T. Therefore at position 31 of the first and third repeats, a specific nucleotide is found for Mg and Mo. The variable site at position 31 of the 94-bp and 65-bp element is indicated in bold and with a * below the position. The repeated elements of *M. incognita* display very different sequence (that cannot be properly aligned) compared with the two other species.

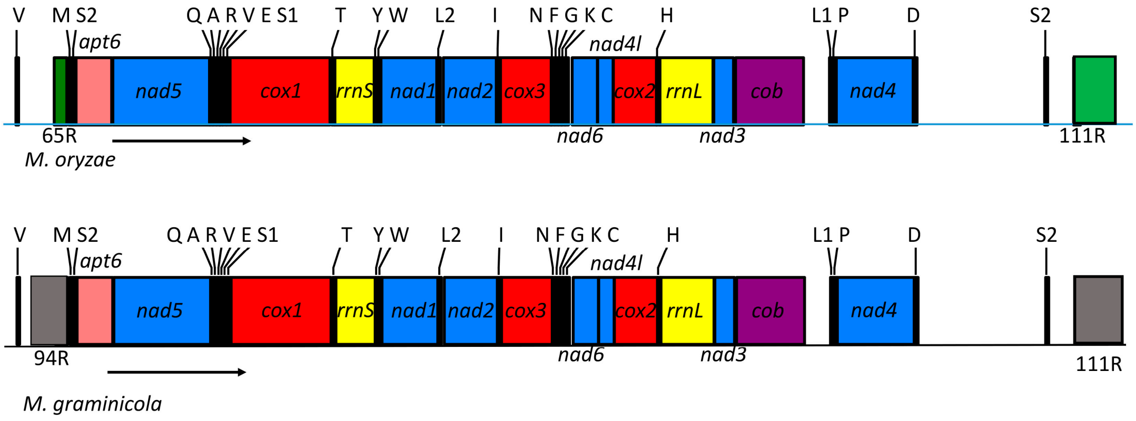

Figure S3. Linearized representation of mitogenomes of *M. oryzae* (this study) and *M. graminicola* (NC_024275.1). Gene and genome size are not drawn to scale. The arrows indicate the direction of the transcription for all genes. The tRNAs are shown by single-letter abbreviation (on the tick marks; see also Besnard et al. [2]).

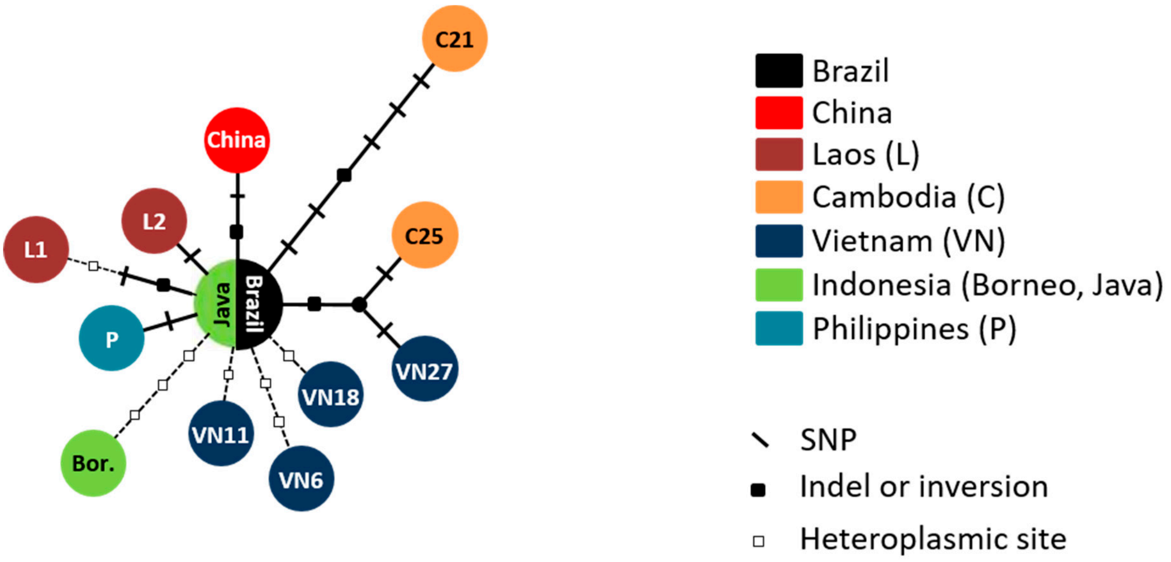

Figure S4. Reduced-median network of *Meloidogyne graminicola* mitochondrial haplotypes including the information of heteroplasmic sites. The network was reconstructed with Network v.5 [3], using the 13 available nematode mitogenome sequences, excluding the 111R region. The nematode populations are indicated in the circles and their geographic origin is displayed by different colors. The number of mutations is shown on the branches with slashes, black squares and white squares that respectively indicate SNPs, indels/inversion and heteroplasmic sites.

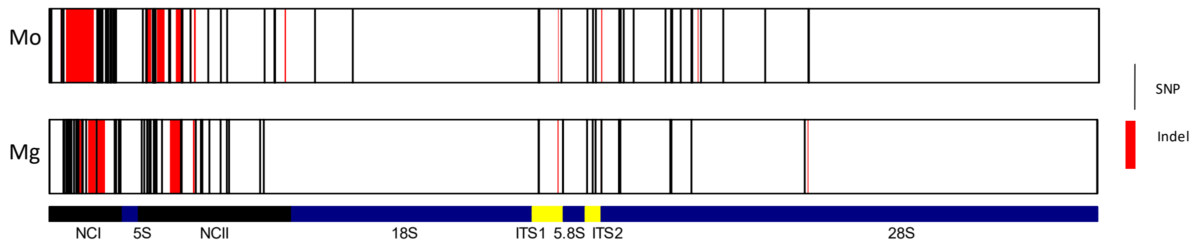

110

Figure S5. Distribution of sequence polymorphisms (SNP or indel) between ribotype I and II along the whole nrDNA cluster of *M. graminicola* (Mg) and *M. oryzae* (Mo)

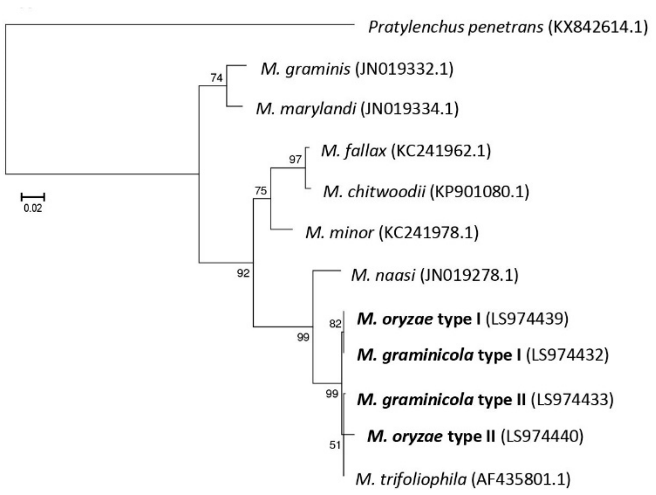

Figure S6. Phylogenetic relationships within *Meloidogyne* species based on ITS using the GTR + G model. Bootstrap values greater than 50% are given on appropriate clades in the Maximum Likelihood tree presented. GenBank sequences are indicated in parenthesis, and sequences from this study are in bold font. The phylogeny is rooted with *Pratylenchus penetrans* as an outgroup. Scale bar represents 0.02 substitutions per nucleotide position.

Table S1. Origin and genome sequencing data summary generated in this study for each nematode isolate

Isolates	Abbreviation	Origin	Number of paired-end reads	Number of cleaned paired-end reads	Read length (bp)	MtDNA sequence depth*	Mitogenome size without 111R (bp)
Meloidogyne graminicola VN6	Mg-VN6	Vietnam	8,187,318	7,591,266	100	1,632× ± 390	`16,809
Meloidogyne graminicola VN11	Mg-VN11	Vietnam	8,613,889	7,873,320	100	1,885× ± 477	16,810
Meloidogyne graminicola VN18	Mg-VN18	Vietnam	9,191,522	8,397,190	100	1,951× ± 513	16,810
Meloidogyne graminicola VN27	Mg-VN27	Vietnam	8,149,454	7,568,959	100	1,742× ± 370	16,905
Meloidogyne graminicola L1	Mg-L1	Laos	6,903,524	6,367,740	125	3,772× ± 764	16,810
Meloidogyne graminicola L2	Mg-L2	Laos	7,639,080	NA	125	197× ± 93	16,811
Meloidogyne graminicola C21	Mg-C21	Cambodia	8,728,853	7,295,797	125	1,571× ± 533	16,813
Meloidogyne graminicola C25	Mg-C25	Cambodia	13,173,663	NA	125	462× ± 211	16,905
Meloidogyne graminicola Java2	Mg-Java	Indonesia	13,657,194	NA	125	74× ± 34	16,811
Meloidogyne graminicola Borneo	Mg-Borneo	Indonesia	8,448,369	NA	125	836× ± 95	16,808
Meloidogyne graminicola P	Mg-P	Philippines	7,762,138	6,146,083	100	1,506× ± 355	16,811
Meloidogyne graminicola Brazil	Mg-Brazil	Brazil	8,440,215	7,347,384	100	1,832× ± 389	16,811
Meloidogyne oryzae M1	Mo-M1	French Guiana	8,559,374	7,900,790	100	794× ± 207	17,069
Meloidogyne oryzae M2	Mo-M2	Suriname	8,316,341	7,662,197	100	1,363× ± 298	17,066

*sequencing depth for the mitogenome assembly with standard deviation

Table S2. Accession numbers of mitochondrial genes of *M. hapla* and *M. floridensis*

Genes	*M. hapla*[1]	*M. floridensis*[2]
cox1	ABLG01002664.1	nMf.1.0.scaf04464
rrnS	ABLG01002664.1	nMf.1.0.scaf04464
cox3	BM884076.1	nMf.1.0.scaf14978
nad4L	-	nMf.1.0.scaf14978
nad3	L76262.1	nMf.1.0.scaf13075
nad4	-	nMf.1.0.scaf13075
nad5	ABLG01002664.1	nMf.1.0.scaf04464

[1]For *M. hapla*, the accession number of genes/contigs were retrieved from GenBank using Nematode BLAST Server (http://xyala.cap.ed.ac.uk/services/blastserver/)

[2]For *M. floridensis*, the scaffolds containing mitochondrial genes were searched using BLAST in the 959 nematode genome database (http://xyala.cap.ed.ac.uk/downloads/959-nematodegenomes/blast/blast.php)

Table S3. Diversity of mitochondrial genomes (excluding 111R) between and within the *Meloidogyne* species

Species	Number of SNPs	Number of indels and inversion
M. graminicola (Mg-P) vs. *M. oryzae* (Mo-M2)	619	58
Between two *M. oryzae* isolates	6	5
Among 13 *M. graminicola* isolates*	11	4

* including a Chinese isolate (KJ139963)

Table S4. Position of SNPs, indels and heteroplasmic sites within the *M. graminicola* mitogenome among 13 isolates

	Position site (location)*																						
Isolate	15743 (NCR)	16549 (NCR)	16724 (NCR)	16726 (NCR)	16831-16833 (NCR)	17099 (NCR)	17325 (NCR)	17486 (NCR)	17512 (NCR)	17774 (*atp6*)	17883 (*atp6*)	18163 (*atp6*)	19441 (*nad5*)	789 (*cox1*)	912 (*cox1*)	1245 (*cox1*)	2386 (*nad1*)	4144 (*cox3*)	6728 (*rrnL*)	8632 (*cob*)	9224 (*nad4*)	10642 (NCR)	11809 (NCR)
Mg-VN6										R (A76; G24)													R (81A; 19G)
Mg-VN11																						Y (T66; C34)	
Mg-VN18																R (74G; 26A)							
Mg-VN27		T					Indel 94R																
Mg-L1					inversion TCC										R (57A; 43G)				C				
Mg-L2				A																			
Mg-C21	indel AT							A	T		T							G			G		
Mg-C25							Indel 94R										G						
Mg-Borneo			K (G53; T47)			K (G54; T46)														Y (T58; C42)			
Mg-Java																							
Mg-P (NC_024275)														C									
Mg-Brazil																							
Mg-China (KJ139963.1)												Indel TA	T										
Consensus sequence	-	C	T	C	GGA	G	-	T	C	G	C	-	A	T	A	A	A	A	T	T	A	C	A
Silent/non silent mutation**	-	-	-	-	-	-	-	-	-	silent	silent	Phe->Leu2	Leu2 -> Phe	Pro->Ser2	Asn->Asp	Ser->Gly	Thr->Ala	Asn->Ser1	-	Phe->Ser2	silent	-	-

The 111R from each population was removed before to proceed to alignment. Therefore, positions 1 and 16,910 of the proposed alignment correspond respectively to the nucleotide number 15,668 and 12,449 from the mitochondrial circular reference genome (NC_024275.1 [2]). The list was established without consideration of variation in mononucleotide stretches (poly A and poly T). The percent of each nucleotide variant for a heteroplasmic position is given in parenthesis.

* in *M. graminicola*-NC_024275

** for protein-coding gene only

Table S5. Assessment of the assembly completeness of available *Meloidogyne graminicola* genome and transcriptome. This analysis was performed with BUSCO v.3 (Benchmarking Universal Single-Copy Orthologs [4]). The BUSCO dataset "Eukaryota *odb9*", which includes 303 Eukaryote single-copy orthologs, was used as the reference.

	Draft genome [5]	Transcriptome [6]
Assembly size (Mb)	38.18	61.08
Number of scaffolds/transcripts	4,304	66,396
N50 value (kb)	20.4	0.4
Complete (%)	73.6	88.1
Complete and single-copy (%)	72.6	35.0
Complete and duplicated (%)	1.0	53.1
Fragmented (%)	15.2	7.6
Missing (%)	11.2	4.3

Table S6. Sequence identity (1 – *p*-distance, in %) between each type of homolog for the three nuclear regions investigated: **A**) nuclear ribosomal DNA (nrDNA) cluster (nucleotide alignment of 7,932 bp), **C**) TAA6 region (nucleotide alignment of 6,546 bp), and **D**) ACC6 region (nucleotide alignment of 6,473 bp). Mg = *Meloidogyne graminicola*; Mo = *M. oryzae*. Intraspecific sequence comparisons are given in green and blue, for Mg and Mo, respectively. Interspecific comparisons are in black

A)

**nrDNA**	Mg Type II	Mo Type I	Mo Type III
Mg Type I	99.29%	**100.00%***	99.25%
Mg Type II		99.38%	98.99%
Mo Type I			99.34%

B)

**TAA6**	Mg Type II	Mo Type I	Mo Type III
Mg Type I	98.36%	**99.98%***	98.06%
Mg Type II		98.38%	97.84%
Mo Type I			98.07%

C)

**ACC6**	Mg Type II	Mo Type I	Mo Type III	Mo Type IV
Mg Type I	97.61%	**99.95%***	98.12%	97.79%
Mg Type II		97.62%	97.65%	97.99%
Mo Type I			98.14%	97.81%
Mo Type III				97.91%

* Note that sequences of Type I from *M. graminicola* and *M. oryzae* are almost identical for the three genomic regions

Table S7. Sequencing depth (with standard deviation) of each type of homolog for the nuclear genomic regions TAA6 and ACC6 among 12 *M. graminicola* and two *M. oryzae* isolates. The size of each sequence type (in bp) is also given for each species.

**Taxa**	**TAA6**			**ACC6**			
	**Type I**	**Type II**	**Type III**	**Type I**	**Type II**	**Type III**	**Type IV**
M. graminicola	**(6,361 bp)**	**(6,399 bp)**	**-**	**(6,151 bp)**	**(6,310 bp)**	**-**	**-**
- Mg-VN6	16.4× ± 5.1	16.5× ± 5.9	-	19.2× ± 4.6	18.8× ± 4.5	-	-
- Mg-VN11	18.4× ± 5.7	18.2× ± 5.3	-	20.8× ± 4.1	20.4× ± 4.9	-	-
- Mg-VN18	20.6× ± 6.2	21.7× ± 7.0	-	21.5× ± 5.0	21.9× ± 4.8	-	-
- Mg-VN27	16.4× ± 4.4	17.4× ± 5.2	-	19.4× ± 5.4	19.6× ± 4.9	-	-
- Mg-L1	22.2× ± 6.7	21.7× ± 7.7	-	19.9× ± 5.2	19.9× ± 5.7	-	-
- Mg-L2	10.2× ± 4.7	9.5× ± 4.1	-	11.6× ± 3.5	9.4× ± 2.9	-	-
- Mg-C21	20.9× ± 6.9	20.8× ± 7.2	-	19.9× ± 4.9	20.3× ± 4.9	-	-
- Mg-C25	12.7× ± 5.6	13.6× ± 6.6	-	14.8× ± 4.1	13.7× ± 3.7	-	-
- Mg-Java	1.3× ± 1.3	1.7× ± 1.4	-	0.9× ± 1.0	0.9× ± 1.0	-	-
- Mg-Borneo	11.6× ± 4.0	12.0× ± 4.1	-	9.4× ± 2.9	10.0× ± 3.2	-	-
- Mg-P	14.1× ± 4.6	14.3× ± 4.6	-	15.4× ± 4.2	14.3× ± 4.0	-	-
- Mg-Brazil	16.6× ± 4.8	17.3× ± 4.5	-	17.4× ± 4.5	17.4× ± 4.6	-	-
M. oryzae	**(6,357 bp)**	**-**	**(6,367 bp)**	**(6,153 bp)**	**-**	**(6,317 bp)***	**(6,309 bp)**
- Mo-M1	11.2× ± 4.3	-	24.2× ± 5.8	11.6× ± 3.1	-	22.9× ± 4.5	-
- Mo-M2	19.2× ± 6.3	-	11.8× ± 4.0	10.6× ± 4.1	-	11.0× ± 3.5	10.1× ± 3.0

* Excluding a repeated element (1,032 bp) in Mo-M2

References

[1] Vurture, G.W.; Sedlazeck, F.J.; Nattestad, M.; Underwood, C.J.; Fang, H.; Gurtowski, J.; Schatz, M.C. GenomeScope: fast reference-free genome profiling from short reads. *Bioinformatics* **2017**, *33*, 2202–2204. doi:10.1093/bioinformatics/btx153.

[2] Besnard, G.; Jühling, F.; Chapuis, É.; Zedane, L.; Lhuillier, É.; Mateille, T.; Bellafiore, S. Fast assembly of the mitochondrial genome of a plant parasitic nematode (*Meloidogyne graminicola*) using next generation sequencing. *C. R. Biol*. **2014**, *337*, 295–301. doi:10.1016/j.crvi.2014.03.003.

[3] Bandelt, H.J.; Forster, P.; Röhl, A. Median-joining networks for inferring intraspecific phylogenies. *Mol. Biol. Evol.* **1999**, *16*, 37–48. doi:10.1093/oxfordjournals.molbev.a026036.

[4] Simao, F.A.; Waterhouse, R.M.; Ioannidis, P.; Kriventseva, E.V.; Zdobnov, E.M. BUSCO: Assessing genome assembly and annotation completeness with single-copy orthologs. *Bioinformatics* **2015,** *31*, 3210–3212. doi:10.1093/bioinformatics/btv351.

[5] Somvanshi, V.S.; Tathode, M.; Shukla, R.N.; Rao, U. Nematode genome announcement: A draft genome for rice root-knot nematode, *Meloidogyne graminicola*. *J. Nematol*. **2018**, *50*, 111–116. doi:10.21307/jofnem-2018-018.

[6] Petitot, A.S.; Dereeper, A.; Agbessi, M.; da Silva, C.; Guy, J.; Ardisson, M.; Fernandez, D. Dual RNA-seq reveals *Meloidogyne graminicola* transcriptome and candidate effectors during the interaction with rice plants. *Mol. Plant Pathol.* **2016**, *17*, 860–874. doi: 10.1111/mpp.12334.
